# From Endometriosis to Encapsulation: A Case Report of Endometriosis Causing Sclerosing Encapsulating Peritonitis

**DOI:** 10.1155/crog/5805116

**Published:** 2026-02-13

**Authors:** Lexi Frankel, Kristen Stearns, Anna Kuan-Celarier, David Childs, Janelle Moulder

**Affiliations:** ^1^ Department of Obstetrics and Gynecology, Wake Forest Baptist Medical Center, Winston-Salem, North Carolina, USA, wakehealth.edu; ^2^ Department of Radiology, Wake Forest Baptist Medical Center, Winston-Salem, North Carolina, USA, wakehealth.edu

**Keywords:** ascites, endometriosis, sclerosing encapsulating peritonitis, spontaneous bacterial peritonitis

## Abstract

Sclerosing encapsulating peritonitis is a rare complication of endometriosis that has sparsely been described in the literature. It involves the development of a thick gray‐white fibrotic membrane, which partially or completely encases the small bowel and can spread intraperitoneally to involve surrounding organs, leading to significant morbidity and mortality. This case describes a 33‐year‐old female with long‐standing, biopsy‐proven stage IV endometriosis who developed recurrent bloody ascites and sclerosing encapsulating peritonitis, leading to multiple hospitalizations for infectious complications and ultimately requiring extensive surgical intervention. This case exemplifies the diagnostic challenge posed by endometriosis‐related ascites, which can mimic malignancy, and highlights the need for awareness of atypical presentations, multidisciplinary management, and individualized surgical decision‐making in advanced disease.

## 1. Introduction

Sclerosing encapsulating peritonitis (SEP) is a rare complication of endometriosis [1, 2]. It involves the development of a thick gray–white fibrotic membrane, which partially or completely encases the small bowel [3]. It can spread intraperitoneally to involve surrounding organs including the large and small intestine, liver, and stomach [4]. The etiology of primary SEP is debated and includes several theories such as retrograde menstruation with superimposed viral infection, retrograde peritonitis, and cell‐mediated immunological tissue damage in response to infection [5–7]. There are other rare causes of secondary SEP in addition to endometriosis, including abdominal surgery, subclinical primary viral, bacterial, or fungal peritonitis, recurrent peritonitis, and malignancy [4]. Although a cause of severe morbidity, encapsulating peritonitis secondary to endometriosis has rarely been described in the literature [4, 8, 9].

## 2. Case Presentation

The patient is a 33‐year‐old G2P0020 with a long‐standing history of endometriosis, initially diagnosed during a diagnostic laparoscopy performed for dysmenorrhea, heavy menstrual bleeding, and cyclic dyschezia. She had no other prior abdominal surgeries. Intraoperative findings at that time were consistent with Stage II endometriosis, demonstrating significant adhesions involving the left ovary and posterior uterus, as well as disease affecting the left fallopian tube. No upper abdominal involvement was identified. She was intermittently treated with norethindrone acetate; however, there were prolonged intervals during which she was not on any form of suppression.

She later re‐presented with worsening pelvic pain and increasing abdominal fullness that she described as distinct from her baseline endometriosis‐related symptoms. At that time, she was not receiving medical suppression. Pelvic ultrasound revealed ascites, presumed to be progressive and longstanding in nature, and she required multiple paracenteses for recurrent bloody fluid accumulation. Cytologic evaluation was negative for malignancy, and endometriosis was considered the most likely etiology.

She subsequently underwent diagnostic laparoscopy with findings consistent with Stage IV endometriosis with endometriotic implants on the right diaphragm, liver, anterior cul‐de‐sac, and bilateral pelvic sidewalls. The posterior cul‐de‐sac was completely obliterated, and bilateral ovaries were appreciably enlarged and adherent to the uterus and sigmoid colon. Cytology studies demonstrated reactive mesothelial cells without evidence of malignancy.

The patient was started on leuprolide acetate and achieved a reduction in her pelvic pain for the next year. She then self‐discontinued the medication and experienced symptom recurrence with reaccumulation of ascites necessitating further paracenteses and hospitalization.

**Figure 1:**
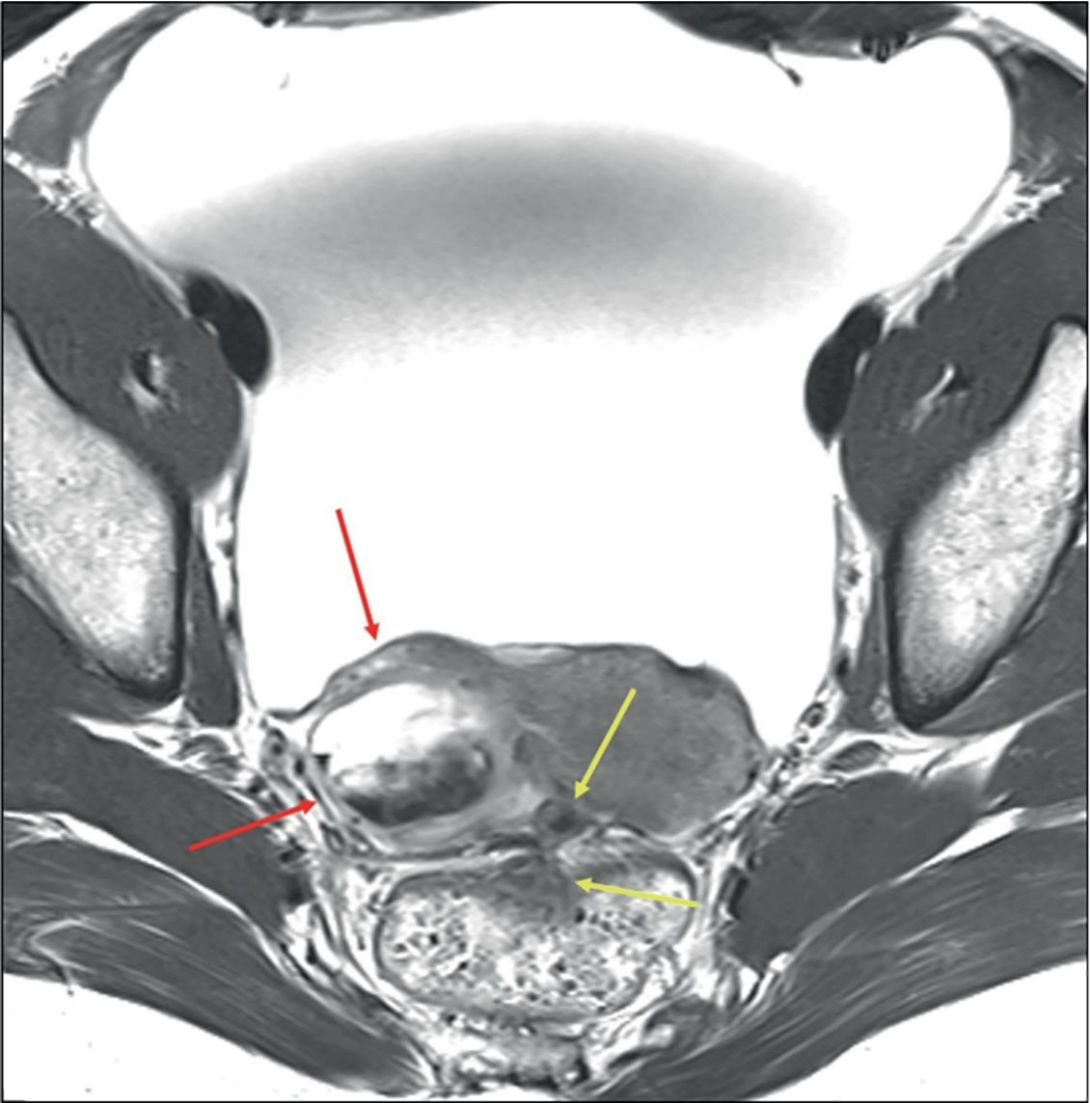
Initial axial T2‐weighted fast spin echo MRI image with findings of endometriosis, including a posterior and medially displaced right ovary with an associated endometrial cyst (red arrows) and deeply infiltrative disease (yellow arrows) obliterating the posterior cul‐de‐sac. Large volume ascites is also noted.

**Figure 2:**
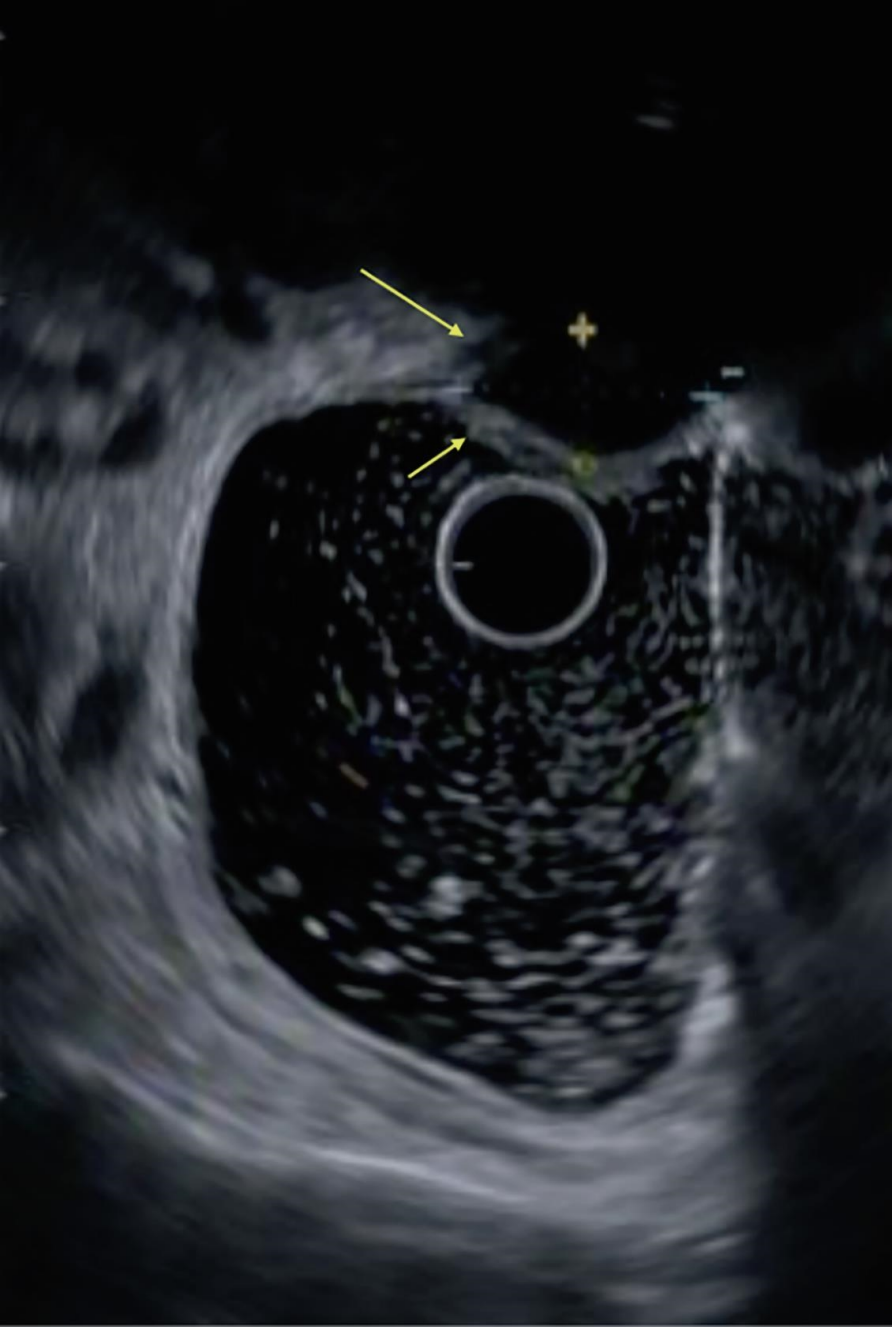
Rectal ultrasound demonstrating a 1.9 cm by 1.0 cm endometrial implant tethering the muscularis propria in the mid‐rectum without rectal invasion.

MRI revealed deep infiltrating endometriosis with obliteration of the posterior cul‐de‐sac, dense tethering of the anterior wall of the rectum, medialization of the right adnexa along the posterior and superior aspect of the uterine corpus, multiple endometriomas and endometrial deposits, and a new periumbilical endometrial deposit (Figure 1). A rectal US showed a 1.9 cm by 1.0 cm endometrial implant resulting in tethering of the muscularis propria in the mid‐rectum but was negative for overt invasion (Figure 2).

**Figure 3:**
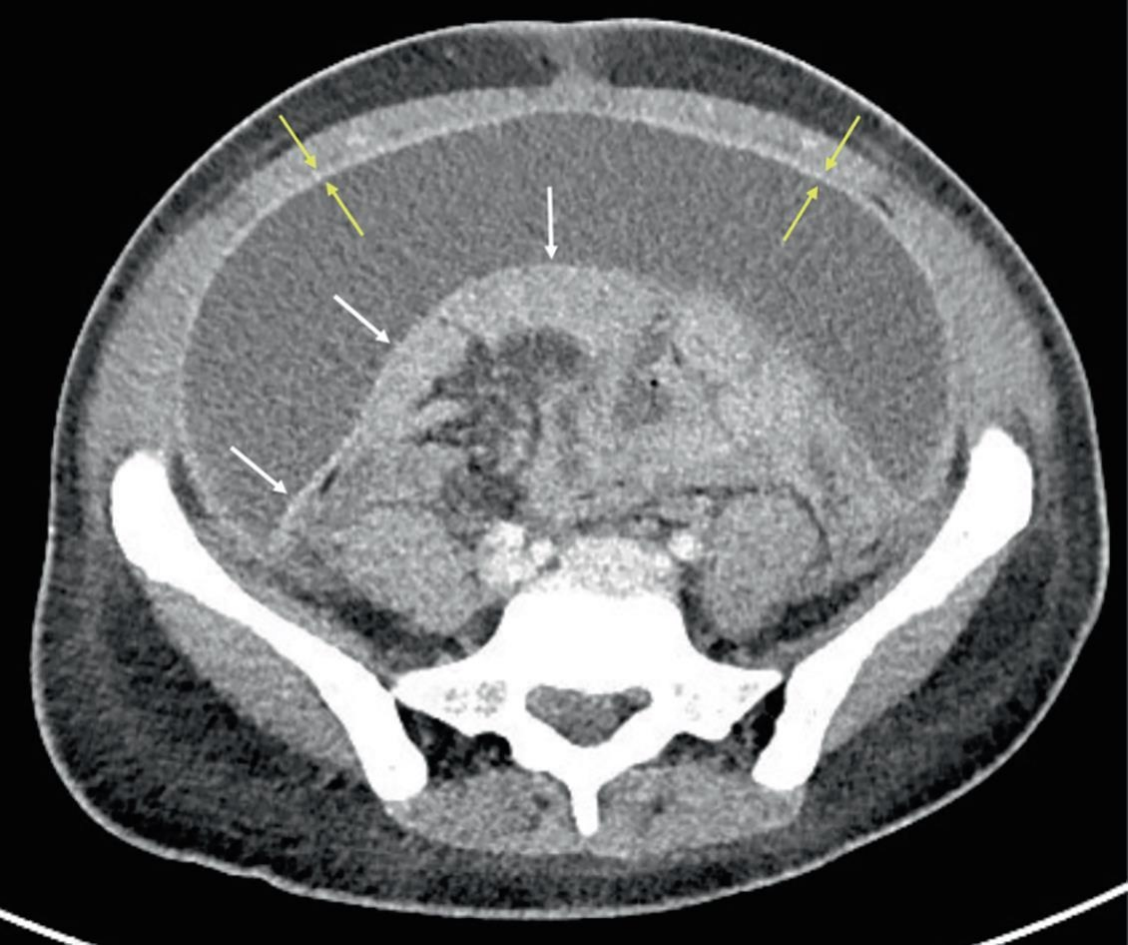
Contrast‐enhanced CT revealing thickening of parietal peritoneum (yellow arrows), and development of coalescent sheet‐like fibrotic tissue (white arrows) surrounding the entire bowel and mesentery, forming a “cocoon” in the central abdomen.

The patient was seen several times in the subsequent months for episodes of spontaneous bacterial peritonitis (SBP). During her fifth presentation with SBP, CT imaging demonstrated findings suggestive of a developing partial small bowel obstruction with external compression of bowel loops by large‐volume tense ascites. There was concern for underlying enteritis and colitis as well as diffuse peritoneal enhancement consistent with SEP (Figure 3). The patient underwent radiology‐guided pelvic drain placement, and culture data revealed candidal peritonitis, prompting initiation of antifungal therapy. She was restarted on leuprolide acetate and norethindrone acetate.

Given the concern on cross‐sectional imaging for interval development of SEP with possible bowel obstruction, the patient underwent exploratory laparotomy. Surgery included extensive lysis of adhesions, total abdominal hysterectomy, salpingo‐oophorectomy, appendectomy, and excision of abdominal wall endometriosis. Her postoperative course was notable for a prolonged ileus, which resolved with nasogastric tube decompression. The patient was discharged home on postoperative day 14. She was initiated on hormone replacement therapy.

## 3. Discussion

SEP is a rare but severe complication of advanced endometriosis, characterized by the formation of a dense fibrocollagenous membrane encasing the bowel, often leading to recurrent bloody ascites, intestinal obstruction, and a clinical picture that can mimic malignancy [3, 4]. SEP can mimic malignancy as it presents with nonspecific symptoms such as abdominal pain, distension, and bowel obstruction, and imaging often shows a thick fibrous membrane encasing bowel loops, ascites, and peritoneal nodularity—features that closely resemble peritoneal carcinomatosis or other intra‐abdominal malignancies [10, 11].

While many cases of SEP are diagnosed at the time of laparotomy, a preoperative diagnosis is possible through ultrasound and computed tomography of the abdomen, as in this case [12]. Hallmark features of SEP include the development of a “cocoon” around visceral structures, forming a distinct separation from the peritoneum [12]. These findings can be appreciated during surgery but may also be identified on preoperative imaging. With early diagnosis, some patients may be effectively managed conservatively [13]. Management is challenging and often requires a combination of hormonal suppression (e.g., GnRH analogs such as leuprolide acetate) to induce quiescence, repeated paracenteses for symptomatic relief, and ultimately extensive surgical intervention—including adhesiolysis, hysterectomy, and excision of endometriotic deposits—when bowel obstruction, uncontrolled infection, or refractory symptoms develop [4, 13]. Hormonal suppression in endometriosis‐associated ascites may prevent accumulation of recurrent ascites and subsequently of SEP [4]. Although there is limited literature, we suspect that early diagnosis and suppression reduce the need for major surgery such as hysterectomy, especially vital in women of childbearing age desiring fertility. As a complication of endometriosis, SEP can cause severe morbidity, especially if there is a diagnostic delay [14, 15]. SEP should be considered when a patient with endometriosis presents with recurrent ascites with or without SBP or in patients presenting with bowel obstruction. Likewise, the diagnosis of endometriosis should be considered for patients presenting with SEP without a known etiology.

## Funding

No funding was received for this manuscript.

## Disclosure

The authors have nothing to report.

## Consent

Written consent obtained from the patient for publication of this case report. It is available upon request.

## Conflicts of Interest

The authors declare no conflicts of interest.

## Data Availability

The data that support the findings of this study are available from the corresponding author upon reasonable request.
